# Effects and safety of magnetic lidocaine microsphere as novel strategy to targeted long-acting peripheral nerve blocks

**DOI:** 10.1186/s12967-026-08752-4

**Published:** 2026-08-12

**Authors:** Li Qiang, Yu Qian, Zheng Lingxi, Peng Lin, Duan Guangyou, Huang He

**Affiliations:** 1 Department of Anesthesiology, The Second Affiliated Hospital of Chongqing Medical University, Chongqing, 400010 China; 2 Department of Anesthesiology, Affiliated Hospital of Southwest Jiaotong University, the Third People’s Hospital of Chengdu, Chengdu, Sichuan 610031 China

**Keywords:** Magnetic microspheres, Lidocaine, Peripheral nerve block, Sustained-release, Targeted drug delivery, Regional anaesthesia, Postoperative pain, PLGA

## Abstract

**Background:**

Local anesthetics have been a cornerstone in the field of anesthesia for over a century, yet they face ongoing challenges in providing sustained postoperative analgesia. The ideal duration for such analgesia is 48–72 hours, a benchmark that current formulations often fail to meet. There is a recognized need for long-acting local anesthetics that can maintain their presence at the periphery of target nerves, thereby extending their therapeutic window. Despite advancements, traditional methods for prolonging nerve blockade—such as adjuvant addition, nerve catheter placement, and repetitive blocks—each have their limitations. The specific gap is the development of a targeted, long-acting peripheral nerve block that can prolong the duration of action of local anesthetics without significant toxic side effects.

**Methods:**

In this study, we developed magnetic lidocaine microspheres using an ultrasonic W1/O/W2 re‑emulsion method, incorporating nano‑iron oxide (Fe₃O₄) and poly(lactic‑co‑glycolic acid) (PLGA). The microspheres were characterized for morphology, magnetic properties, and in vitro drug release. Pharmacokinetics and pharmacodynamics were evaluated in animal models, including electrophysiological and behavioral assessments of peripheral nerve block and analgesia. Blood concentrations were measured by liquid chromatography‑mass spectrometry (LC‑MS), and drug distribution was observed by MRI. Toxicity was assessed via sciatic nerve pathology and cytotoxicity tests on rat Schwann cells.

**Results:**

Under a static magnetic field, the magnetic lidocaine microspheres prolonged peripheral nerve blockade for up to 60 hours, with the compound action potential amplitude suppressed to <20% of baseline for 48 hours. In vitro release reached 97.3 ± 2.1% at 60 hours with an initial burst of 76% at 24 hours. The formulation exhibited concentration‑dependent cytotoxicity on Schwann cells, with significantly higher cell viability compared to free lidocaine at clinically relevant concentrations (≥250 μM, *p* < 0.01; 500 μM, *p* < 0.05). Pharmacokinetic analysis revealed that the MLM‑M group had a 21.33‑fold longer Tₘₐₓ and a 15.62‑fold longer t₁/₂ compared to free lidocaine, with AUC₀‑₄₈ reduced to approximately 4.5% of that in the free lidocaine groups (0.341 ± 0.034 vs. 7.566 ± 0.302 μg·h/mL), confirming sustained local delivery with minimal systemic exposure.

**Conclusion:**

The magnetic lidocaine microspheres effectively prolonged peripheral nerve blocks under the influence of a static magnetic field, achieving sustained drug release over 60 hours with significantly reduced systemic absorption compared to conventional lidocaine. Notably, this targeted approach demonstrated concentration‑dependent cytotoxicity on Schwann cells and maintained therapeutic concentrations at the nerve site while limiting systemic exposure, as evidenced by pharmacokinetic and MRI analyses. These findings demonstrate that magnetic lidocaine microspheres can achieve prolonged, site-targeted nerve blockade in a preclinical model, with reduced systemic exposure and favorable local tissue compatibility. While these results are encouraging, further studies in postoperative pain models and large-animal studies are needed before clinical translation can be considered. If successfully translated, this technology could potentially offer a new approach to prolonged regional analgesia with reduced reliance on systemic opioids.

**Clinical trial registration:**

This study is not a randomized controlled trial and therefore was not registered in a public clinical trials registry.

## Significance Statement

This study introduces magnetic lidocaine microspheres that provide targeted, sustained peripheral nerve blockade for over 60 hours under a magnetic field, significantly prolonging analgesia while reducing systemic toxicity and offering a safer alternative to opioids for postoperative pain management.

## Introduction

Local anaesthetics, a cornerstone in the field of local anesthesia for over a century, face ongoing challenges in achieving sustained postoperative analgesia. The ideal duration for such analgesia is 48–72 hours, a benchmark that current formulations often fail to meet [1]. To address this, there is an urgent need to develop long-acting local anesthetics that can maintain their presence at the periphery of target nerves, thereby extending their therapeutic window. Traditional methods such as visualization [2], adjuvant addition [3], nerve catheter placement [4], and repetitive blocks each have their limitations. Our study introduces a novel approach: the use of carriers to achieve targeted aggregation and controlled release of local anesthetics, thereby prolonging analgesia. This targeted drug delivery system, initially developed for oncology, offers the advantages of reduced systemic drug accumulation and extended drug efficacy.

We focus on poly(lactic-polyglycolic acid) (PLGA) microspheres, which release drugs through diffusion and degradation mechanisms [5]. Diffusion involves the dissolution of the drug into the microspheres’ voids [6], while degradation refers to the drug’s release as the PLGA shell dissolves [7]. Our study diverges from others by incorporating magnetic microspheres with specific magnetic field strengths for passive targeting and prolonged drug release.

Previous research on local anesthetic retardants has not fully explored the free diffusion and toxicity of these microspheres post-injection. Our study addresses this gap by designing, preparing, and characterizing magnetic lidocaine microspheres for passive targeting. We further investigate their nerve-blocking effects and sustained analgesic potential through animal experiments and assess their toxicity on peripheral nerves using cellular biology techniques.

## Materials and methods

### Materials and reagent of microsphere synthesis

Lidocaine hydrochloride and procaine hydrochloride were purchased from McLean Biochemical Technology Co. (Shanghai, China). Nano-iron oxide (Fe3O4) was purchased from the Aladdin Biochemical Technology Co. (Shanghai, China). Polylactic acid (PLGA; 50:50, Mw = 5000, Mw = 10000, Mw = 15,000) polymers were purchased from Daigang Biological Engineering Co. (Jinan, China). Gelatin was purchased from Sigma–Aldrich (Germany).

Polyvinyl alcohol (PVA) was purchased from Kearon Chemicals Co. (Chengdu, China). Dichloromethane (DCM) and caustic soda (NaOH) were purchased from the Kelong chemical reagent factory (Chengdu, China). Ethyl acetate was purchased from Fuchen Chemical Reagent Co. (Tianjin, China). Methanol was purchased from Jindong Tianzheng Fine Chemical Reagent Factory (Tianjin, China). 4% paraformaldehyde universal tissue fixative was purchased from Lanjieke Technology Co. (Beijing, China). The laboratory provided 25% urethane injection, deionized water, and 0.9% physiological saline.

### Preparation of magnetic lidocaine microspheres

**Fig. 1:**
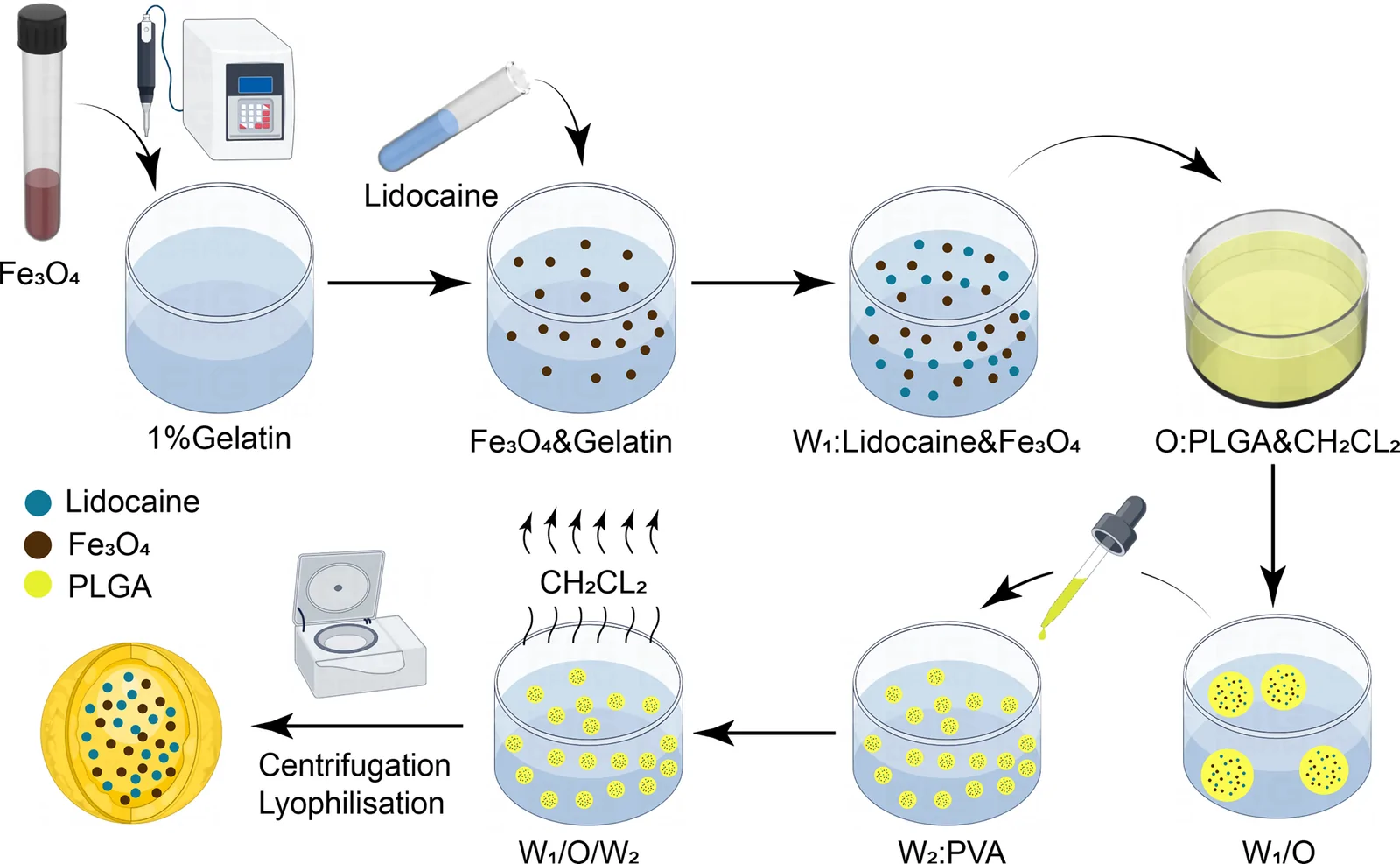
Schematic diagram of the preparation process of magnetic lidocaine microspheres. PLGA, poly (lactic-co-glycolic acid)

Magnetic lidocaine microspheres were prepared by ultrasonic W1/O/W2 Re-emulsion method. A 10% gelatin solution of 10 ml was configured, and 0.1 g of nano iron tetraoxide was added, followed by ultrasonic dispersion for 10 minutes. Then, 2 ml of deionised water was taken, into which 0.1 g of lidocaine hydrochloride standard was added, fully shaken to dissolve, and subsequently dropped into the aqueous ferric oxide gelatin solution to serve as the inner aqueous phase (W1). 0.4 g PLGA was dissolved in 8 ml dichloromethane and fully dissolved as the oil phase (O). The internal aqueous phase and the oil phase were mixed thoroughly and dispersed at high speed for 5 min using a high-speed shear disperser at 8000 r/min to form a stable primary emulsion (W1/O). A 3% (w/v) polyvinyl alcohol (PVA) aqueous solution was prepared as the external aqueous phase (W2). The primary emulsion (W1/O) was then added dropwise (40 drops/min) into the external aqueous phase (W2), and magnetically stirred in a fume hood for 6 hours, and then washed by centrifugation at 2000 r/min for 3 times after the organic solvent evaporated, and lyophilised to obtain the magnetic lidocaine microsphere powder (Fig. 1).

### Characterize particle morphology of magnetic lidocaine microspheres

An appropriate amount of microsphere powder was added to deionized water and ultrasonically dispersed before measurement. The particle morphology was characterized using transmission electron microscopy (TEM, FEi-F20, Fei Co., USA) and scanning electron microscopy (SEM, SU5000, Hitachi Co., Tokyo). The magnetic response parameters of the microspheres under magnetic felds were measured using a vibrating sample magnetometer (VSM, Lakeshore-7404, Lake Shore Co., USA) to obtain the hysteresis curves. In addition, an appropriate amount of magnetic lidocaine microspheres was taken in ethanol and dispersed by ultrasonication for 5 min, and the microsphere particle size was determined by laser particle sizer (Mastersizer 3000^+^, Malvern Panalytical Ltd, UK) and zeta potential analyser (Zeta-Meter 4.0^+,^ Rubolab GmbH Ltd, Germany), and the determination of zeta potential was carried out at pH = 7.4.

### In vitro drug release study

#### Establishment of the standard curve for lidocaine hydrochloride

Lidocaine hydrochloride (10 mg) was dissolved in phosphate bufered saline (PBS) solution to achieve a concentration of 1 mg/mL lidocaine hydrochloride solution.

Lidocaine hydrochloride was diluted into 5, 10, 20, 40, and 80 μg/mL solutions, and the absorbance values at 263 nm were measured by ultraviolet (UV)-visible spectrophotometer to plot the standard curve of lidocaine hydrochloride concentration (C)-absorbance value (A).

#### In vitro release profle of magnetic lidocaine microspheres

In this part, 30 mg of magnetic lidocaine microspheres were weighed precisely and placed in a centrifuge tube containing 30 mL of PBS solution (pH 7.4). The centrifuge tubes was placed on a constant temperature water bath shaker at 37 °C with an oscillation frequency of 100 times/min. The supernatant (2 mL) was collected at 0.5, 1, 2, 4, 8, 12, 24, 48, and 60 h, respectively, and the absorbance was measured at 263 nm using a UV spectrophotometer. Next, 2 mL of PBS was added, and the concentration of lidocaine hydrochloride was calculated according to its standard curve at the corresponding time. The cumulative amount of drug released at each time point was measured, and the in vitro release curve was plotted.

#### Drug loading and encapsulation rate

In this study, 10 mg of magnetic lidocaine microspheres was precisely weighed in a volumetric flask, and 5 mL of Dichloromethane (DCM) was added to dissolve and impair the microspheres. Deionized water was added and centrifuged to remove the shell and Fe3O4, and the volume was fixed. The absorbance was measured at 263 nm using a UV–visible spectrophotometer, and the mass of lidocaine hydrochloride in the solution was calculated using the equation for the standard curve of lidocaine hydrochloride. The drug-loading and encapsulation rates of lidocaine hydrochloride

were calculated using Eqs. (1) and (2):

1 $$LE\% = {{We} \over {Wm}} \times 100\% $$

2 $$EN\% = {{1 - Cf} \over {Ct}} \times 100\% $$

*LE*: percentage of drug loaded in magnetic lidocaine microspheres; *We*: amount of drug encapsulated in PLGA microspheres; *Wm*: total weight of drug-loaded magnetic lidocaine microspheres; *EF*: percentage of drug encapsulated in magnetic lidocaine microspheres; *Cf*: the amount of free drug; *Ct:* total amount of input drug.

### Peripheral nerve block and analgesia experiment

#### Animals

Specific pathogen-free New Zealand rabbits (3000–3500 g, 24, half male and half female) were provided by the animal experiment center at the ChengDu DOSSY Experimental Animals CO,LTD. The rabbits were 5–6 months old and routinely kept at an indoor temperature with unlimited access to food and water. The sample size was determined using GLIMMPSE 3.1.0 software. Based on preliminary electrophysiological data from 3 rabbits, we assumed an effect size (Cohen’s d) of 1.5 for the difference in block duration between MLM‑M and MLM‑NM groups (primary outcome: time to 50% recovery of compound action potential amplitude), with a two‑sided significance level α = 0.05 and power = 0.80. This calculation yielded a minimum of 4 animals per group. To account for potential technical failures, we included 5 animals per group. For behavioral (non‑parametric) outcomes, the same sample size was used as it provided adequate power for detecting clinically relevant differences in median scores. All post hoc comparisons were adjusted for multiple testing using Tukey’s (for parametric data) or Dunn’s (for non‑parametric data) procedures with Bonferroni correction for repeated time points. This calculation accounted for potential variability in electrophysiological and behavioral responses among animals, ensuring sufficient statistical power to detect significant differences between the treatment and control groups. The Institutional Animal Care and Use Committee approved all animal study protocols at the ChengDu DOSSY Experimental Animals CO,LTD (ethical approval number: AEWC-2023–11105-SPF). All animal experiments were conducted in accordance with the ARRIVE guidelines 2.0 and the National Institutes of Health (NIH) Guide for the Care and Use of Laboratory Animals (8th edition, 2011)

#### Establishing the sciatic nerve block model and measurement of the electrophysiologyical properties of the sciatic nerve

Prior to injection, the lyophilized magnetic lidocaine microspheres were reconstituted in sterile saline such that each 2 mL suspension contained 20 mg of lidocaine, based on the measured drug loading of 6.70%, thereby matching the lidocaine dose in the 2 mL of 1% lidocaine solution (20 mg).

Rabbits received an intravenous injection of 25% uratan (5 mL/kg) into their ear margins until their corneal reflexes disappeared. After removing the hair, the skin was incised in the midline of the posterior side of the lower limbs bilaterally and the sciatic nerves were found. After fully exposing the sciatic nerves, the catheter was left next to the sciatic nerve trunks and was fixed properly; the nerve recording electrodes were placed into the distal end of the sciatic nerves, and the stimulation electrodes into the proximal end of the sciatic nerves, and the electrodes were embedded in paraffin wax and then fixed properly. For electrophysiological and behavioral assessments, rabbits were randomly divided into three treatment groups (*n* = 5 per group) and one saline control group (*n* = 5, contralateral side pooled):MLM‑M group: perineural injection of 2 ml magnetic lidocaine microspheres (equivalent lidocaine dose as 1% solution) with an external static magnetic field (120 mT) applied around the injection site.MLM‑NM group: same microsphere injection without the magnetic field. L‑M group: perineural injection of 1% lidocaine (same dose) with the magnetic field.The contralateral sciatic nerve in each rabbit received 2 mL of saline and served as the control (C group).After the injection was completed, magnets were placed in the anterior and posterior sides of the rabbits’ bilateral lower limbs to set up the same static magnetic field. Compound action potentials, threshold strength, maximum stimulus strength and conduction velocity were measured in the sciatic nerve trunks of the rabbits bilaterally before drug injection, and 30 min, 2 h, 8 h, 16 h, 24 h, 48 h, and 60 h after the injection of the drug.

#### Magnetic field application parameters

To achieve targeted aggregation of the magnetic microspheres, immediately after injection, a flexible neodymium (NdFeB) permanent magnet patch (configured in a Halbach array pattern; diameter: 20 mm; thickness: 10 mm) was placed on both the anterior and posterior sides of the injection site (i.e., around the sciatic nerve) in each lower limb of the rabbits. The magnets were coated with epoxy resin to prevent corrosion. Using a gaussmeter (F-41, Quantum Design China, China), it was confirmed that the static magnetic field strength generated by each magnet patch at the target neural site was 120 mT with a gradient of 6 T/m, and the magnetic field oriented perpendicular to the nerve course. The magnets were secured with medical adhesive tape and remained in place throughout the entire 60-hour experimental observation period to ensure consistent and stable application of the magnetic field.

#### Behavioural evaluation

Motor function of the rabbits’ hind limbs was assessed using the Toe-Opening Reflex score (Annex I) at baseline (before drug injection) and at 30 minutes, 2 hours, 8 hours, 16 hours, 24 hours, 48 hours, and 60 hours post-injection.

### Pharmacodynamic and pharmacokinetic studies

#### Animals and sciatic nerve block model

The source of experimental animals and the establishment of sciatic nerve block models were the same as in the previous section, and all sciatic nerve block models were right-sided. Five rabbits received 2 mL of magnetic lidocaine microspheres (reconstituted to contain 20 mg of lidocaine, matching the 2 mL of 1% lidocaine solution) via the perineural catheter with a static magnetic field applied around the injection site (MLM-M group). Another five rabbits received the same microsphere injection without the magnetic field (MLM-NM group). For comparison, five rabbits received 2 mL of 1% lidocaine (the same lidocaine dose as the microsphere formulation) with the magnetic field (L-M group), and five rabbits received the same lidocaine solution without the magnetic field (L-NM group).

#### Blood concentration measurement

Blood was collected in 2 mL from a vein at the ear margin of experimental rabbits at the immediate post-injection, 5 min, 15 min, 30 min, 1 h, 2 h, 3 h, 4 h, 6 h, 8 h, 12 h, 24 h, 36 h, 48 h, and 72 h after injection, and lidocaine blood concentration was determined by Liquid Chromatography-Mass Spectrometer (LC-MS), respectively. Results were analysed using a non-atrial model and the main pharmacokinetic parameters (t_1/2_, C_max_, T_max_, MRT and AUC) were calculated.

#### Drug distribution in vivo

MRI was performed in New Zealand rabbits immediately after drug injection, 15 min, 2 h and 8 h after drug injection to observe the distribution of the drug around the sciatic nerve after drug injection. Of note, for the MRI scans, the external magnets were temporarily removed during image acquisition to prevent magnetic field interference with the MRI scanner, and were repositioned immediately after each imaging session. This temporary removal (lasting < 5 minutes per session) is not expected to significantly alter microsphere distribution, as the retention effect is based on the cumulative exposure to the magnetic field over the observation period.

### Drug toxicity studies

#### Sciatic nerve pathology experiments

Two rabbits from each of the previous MLM-M group and MLM-NM group were humanely euthanized at 3, 7 and 14 days after drug administration, and the injection site, sciatic nerve and surrounding tissues (1 cm^3^) were taken, fixed in 4% paraformaldehyde solution for 48 hours, wax-sealed, sectioned, HE-stained, and the morphology of the sciatic nerve and the microstructure of the tissues around it were observed under the light microscope.

All slides were coded with random numbers by an independent technician. Two board-certified pathologists independently evaluated the slides under blinded conditions using a semi-quantitative scoring system assessing: (i) inflammatory cell infiltration, (ii) perineural fibrosis, (iii) axonal degeneration, and (iv) myelin sheath integrity (each scored 0–3). Ten non-overlapping high-power fields (×400) were assessed per specimen. Inter-rater reliability was assessed using Cohen’s kappa coefficient and intraclass correlation coefficient (ICC, two-way random model, absolute agreement). Discrepancies were resolved by consensus discussion.

#### Cytotoxicity test

The rat Schwann cell line (RSC96) and its culture medium were obtained from Procell Life Science & Technology Co., Ltd. (Wuhan, China). Primary antibodies against S100β and CD90 were purchased from Sanying Biotechnology (Wuhan, China). Type I collagenase was sourced from Thermo Fisher Scientific (Waltham, MA, USA), while forskolin and cytarabine were acquired from MedChemExpress (Monmouth Junction, NJ, USA). The CCK-8 assay kit was procured from Meilun Biotechnology (Dalian, China).Cells were maintained in complete Schwann cell medium at 37 °C in a humidified 5% CO₂ incubator. Upon reaching 90% confluence, they were detached using 0.25% trypsin-EDTA and subcultured at a density of 1 × 10^4^ cells/well in 96-well plates. After 20 hours of pre-incubation, Cells were treated with: free lidocaine (0, 100, 250, 500, 1000, or 2000 μM final concentration); or magnetic lidocaine microspheres at concentrations corresponding to equivalent lidocaine doses (0, 100, 250, 500, 1000, or 2000 μM lidocaine equivalent), based on the measured drug loading of 6.70% (i.e., the amount of microspheres added was calculated to deliver the indicated final lidocaine concentration).Following 72 hours of exposure, 10 μL of CCK-8 reagent was added to each well, and plates were incubated for 3 hours. Absorbance was measured at 450 nm using a microplate reader (Model: SpectraMax i3x, Molecular Devices, San Jose, CA, USA). Cell viability was assessed with at least three technical replicates for each concentration, and the entire experiment was independently repeated three times (*n* = 3).

#### Blinding

Behavioral and electrophysiological assessments were performed by an investigator blinded to the treatment allocation. Pharmacokinetic sample analysis by LC-MS, histological scoring, and CCK-8 assay readings were also conducted by laboratory personnel who were blinded to group assignments. Group unblinding occurred only after all data analyses were completed.

#### Statistical analysis

Data were expressed as mean ± standard deviation (SD) for normally distributed variables or median and interquartile range (IQR) for non-parametric measures. Normality was assessed using the Shapiro-Wilk test. For normally distributed data, statistical comparisons among the four treatment groups (MLM‑M, MLM‑NM, L‑M, and L‑NM) were performed using one-way or two-way ANOVA, followed by post hoc Tukey’s test for multiple comparisons. For non-normally distributed data, the Kruskal‑Wallis test was used for between‑group comparisons at each time point, followed by Dunn’s post hoc test for pairwise multiple comparisons. The Friedman test was applied for within‑group comparisons across time points (repeated measures), followed by appropriate post hoc tests when significant differences were detected. A *p* value < 0.05 was considered statistically significant. All analyses were conducted using GraphPad Prism (version 9.1.0, GraphPad Software, San Diego, CA, USA).

## Results

### Magnetic lidocaine microspheres and their characterisation

**Fig. 2:**
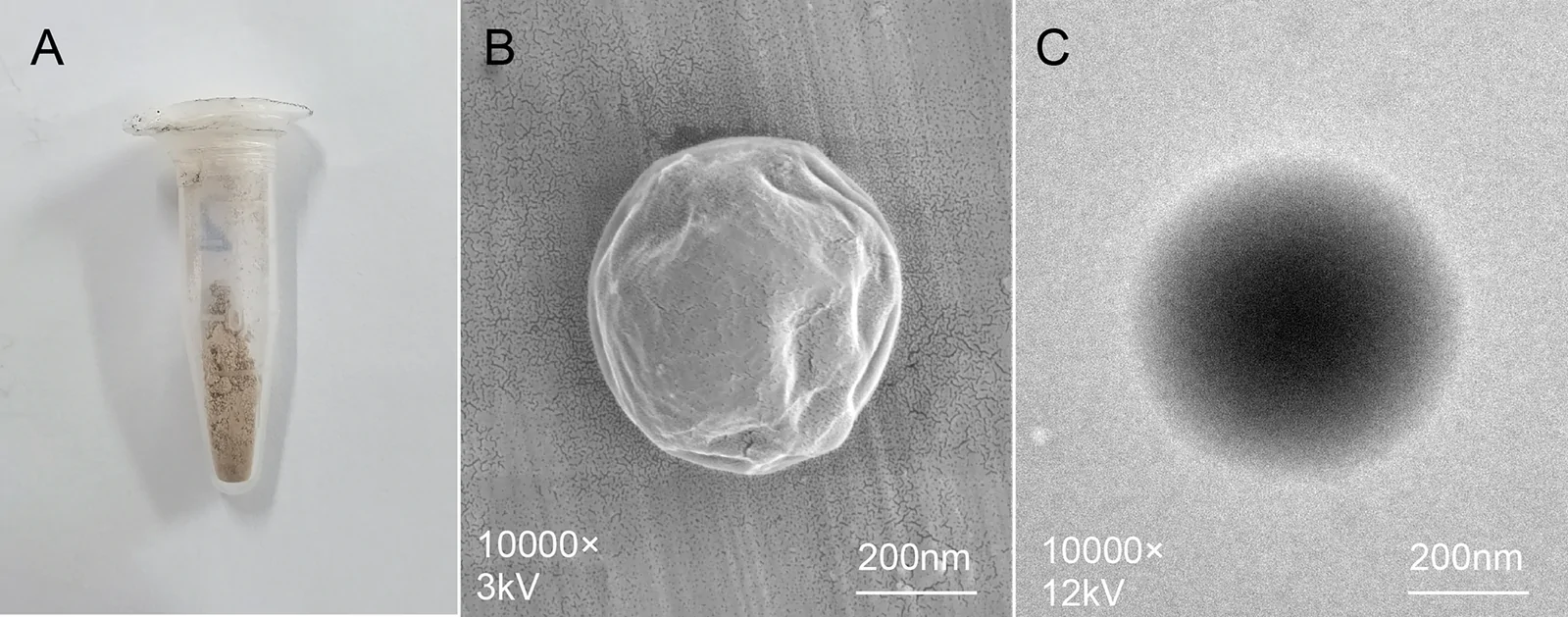
Characterization of magnetic lidocaine microspheres. **A**, synthetic lyophilised powder of magnetic lidocaine microspheres; **B**, magnetic lidocaine microspheres under scanning electron microscopy; **C**, magnetic lidocaine microspheres under transmission electron microscopy

**Fig. 3:**
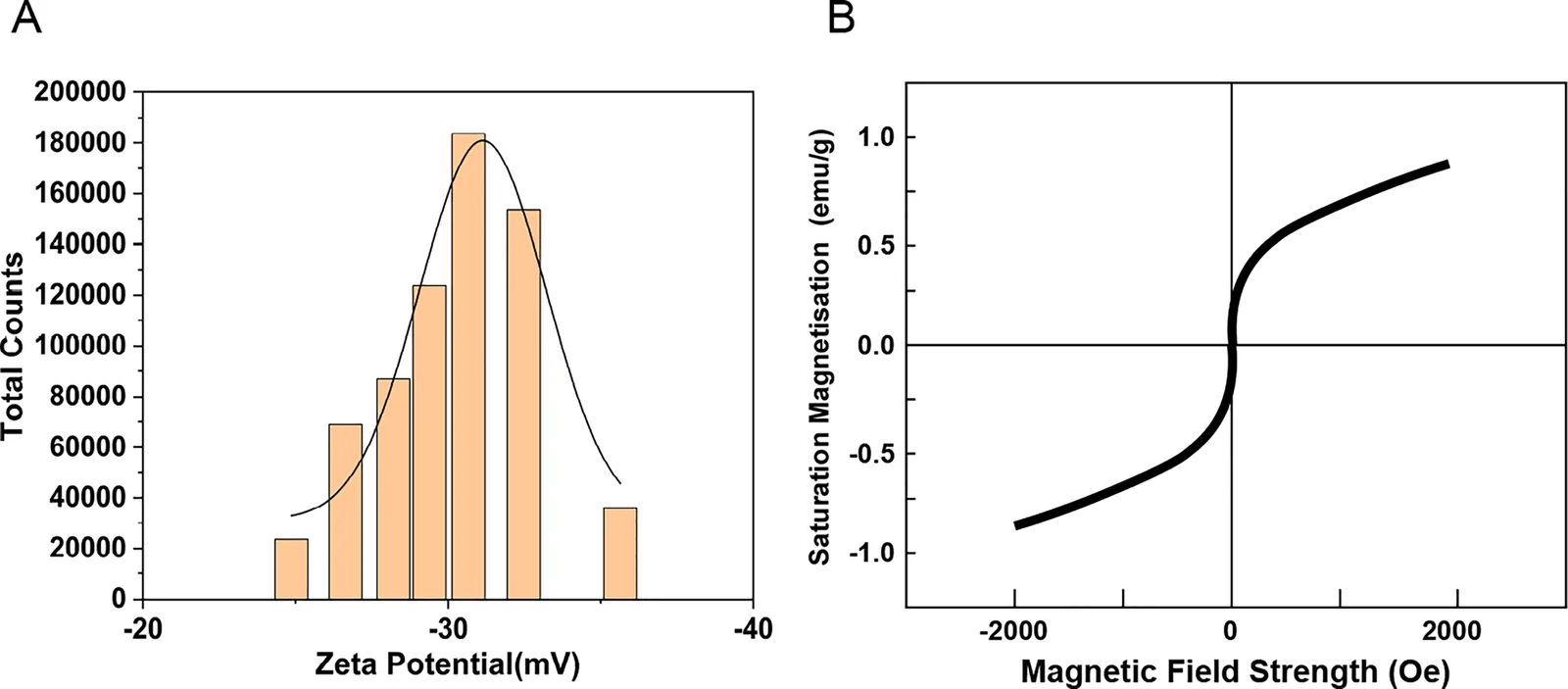
Physicochemical properties of magnetic lidocaine microspheres. **A**, zeta potential of magnetic lidocaine microspheres; **B**, hysteresis lines of magnetic lidocaine microspheres

The magnetic lidocaine microspheres lyophilised powder prepared in this experiment was brown (Fig. 2, A). The magnetic lidocaine microspheres were observed under scanning electron microscope as monodisperse, regular spherical shape (Fig. 2, B), and under transmission electron microscope, the outer PLGA shell of the magnetic lidocaine microspheres, as well as the inner encapsulation of the drug and the tetroso iron nanoparticles were visible (Fig. 2, C). The diameter of lidocaine microspheres was measured to be (435.27 ± 31.82) nm with a zeta potential of −31.14 ± 0.27 mV (Fig. 3, A).

The zeta potential distribution of magnetic lidocaine microspheres, measuring −31.14 ± 0.27 mV at physiological pH (7.4), indicating good colloidal stability due to the strong negative surface charge (Fig. 3.A). The hysteresis line of magnetic lidocaine microspheres was S-shaped at room temperature and under the condition of applied positive and negative magnetic field (Fig. 3, B), and the saturation magnetisation intensity was about 0.9–1.0 emu/g. In the absence of applied magnetic field, when the magnetic field intensity was 0, the hysteresis line passed through the origin. Therefore, the coercivity (Hcm) = residual magnetisation strength (M)=0 and no hysteresis is generated, indicating that the magnetic lidocaine microspheres has good superparamagnetic properties at room temperature.

### Sustained release of magnetic lidocaine microspheres in vitro

**Fig. 4:**
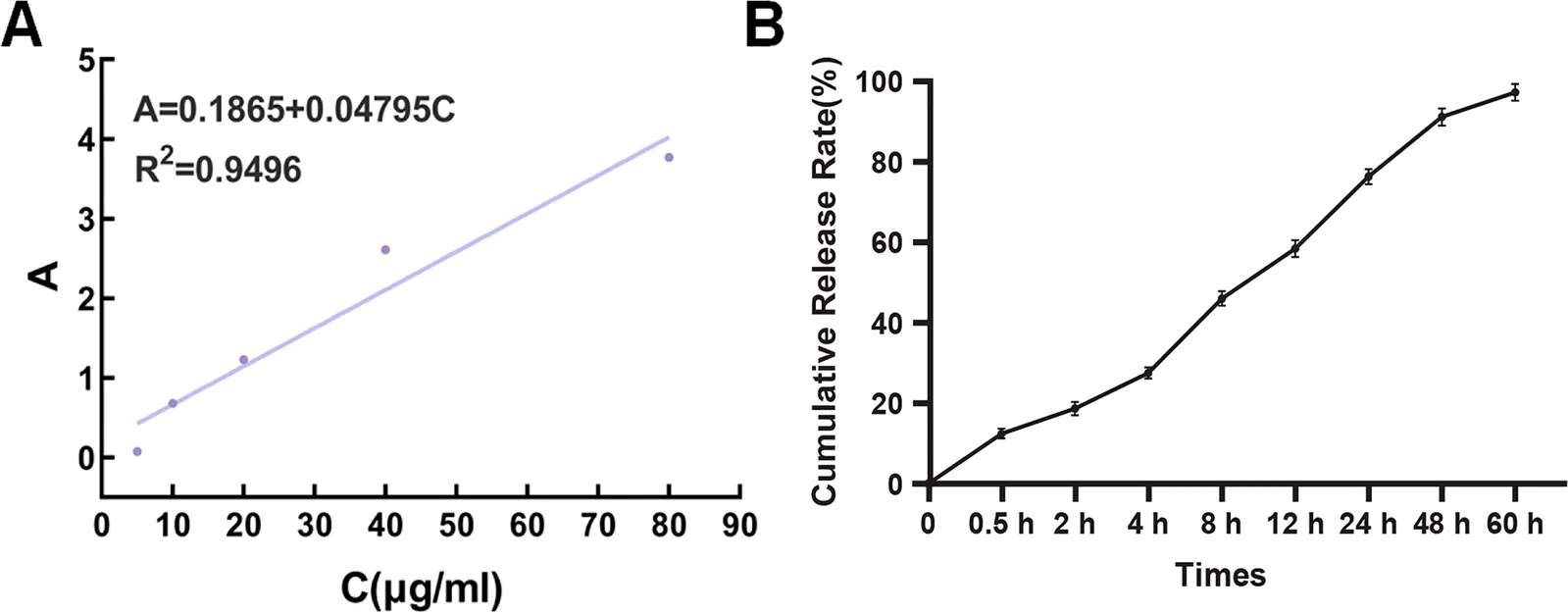
In vitro release assay. **A**, concentration-absorbance standard curve of lidocaine hydrochloride solution. **B**, in vitro release curve of magnetic lidocaine microspheres

Figure 4Bshows the in vitro release profile of magnetic lidocaine microspheres (mean ± SD, *n* = 3 independent batches). At 60 h, the cumulative release of lidocaine hydrochloride was 97.3 ± 2.1%. The release curve showed that more lidocaine was released at 24 h. The concentration (C)-absorbance (A) standard curve of lidocaine hydrochloride solution (Fig. 4A) was converted to show that the encapsulation rate of magnetic lidocaine microspheres was 47.31% and the drug loading was 6.70%.

### The effect of magnetic lidocaine microspheres on the electrophysiology of the sciatic nerve

**Fig. 5:**
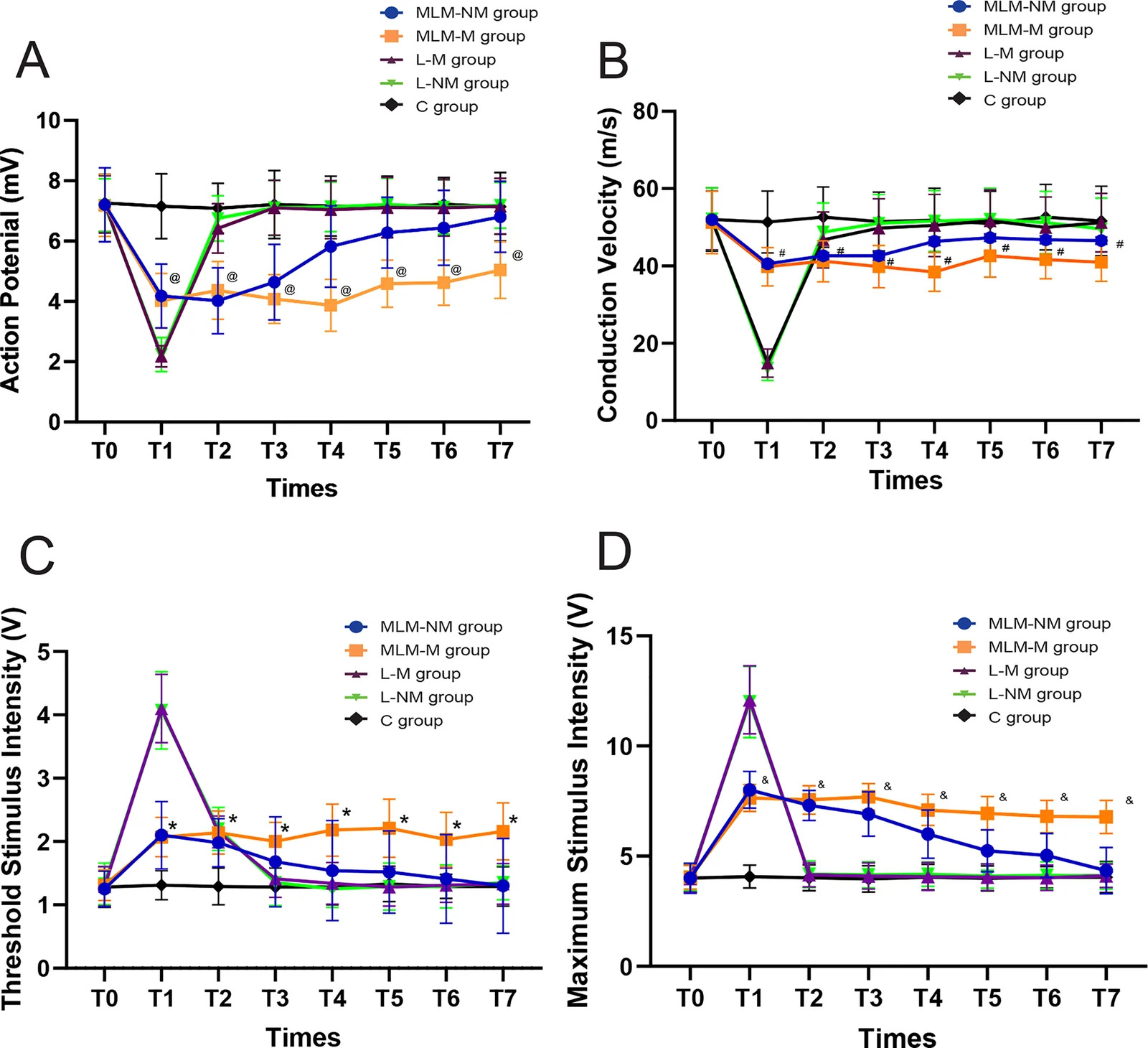
Electrophysiological properties of the rabbit sciatic nerve following perineural injection of magnetic lidocaine microspheres (MLMs) and control agents. (**A**–**D**) time-dependent changes in compound action potential amplitude (**A**), nerve conduction velocity (**B**), threshold stimulus intensity (**C**), and maximal stimulus intensity (**D**). Measurements were performed at baseline (T0, before injection) and at 0.5 h (T1), 2 h (T2), 8 h (T3), 16 h (T4), 24 h (T5), 48 h (T6), and 60 h (T7) post-injection. Rabbits were randomly assigned to four groups (*n* = 5 per group): MLM-M group (magnetic lidocaine microspheres + static magnetic field, 120 mT), MLM-NM group (magnetic lidocaine microspheres without magnetic field), L-M group (1% lidocaine solution + static magnetic field), L-NM group (1% lidocaine solution without magnetic field) and **C** group (contralateral side, 2 mL saline, no magnetic field). Data are presented as mean ± SD. Statistical comparisons among groups at each time point were performed using two-way ANOVA followed by Tukey’s post hoc test. Key statistical results are described in the main text

To evaluate the individual contributions of the magnetic field and the microsphere encapsulation, we compared the electrophysiological parameters among the MLM‑M, MLM‑NM, L‑M and L-NM groups (Fig. 5A‑D).

As shown in Fig. 5A, the action potential amplitude in the MLM‑M group dropped to less than 20% of baseline within 2 hours and remained significantly suppressed for up to 60 hours. In contrast, the MLM‑NM group (microspheres without magnetic field) recovered to 80% of baseline by 24 hours, and the L‑M group (free lidocaine with magnetic field) recovered by 8 hours. The differences between MLM‑M and MLM‑NM were statistically significant from 16 to 60 hours (*p* < 0.01), and between MLM‑M and L‑M from 8 to 60 hours (*p* < 0.001). Similar patterns were observed for nerve conduction velocity (Fig. 5B), threshold stimulus intensity (Fig. 5C), and maximal stimulus intensity (Fig. 5D). The L-NM group showed no significant difference from the L-M group at any time point (*p* > 0.05).

These results demonstrate that both the external magnetic field and the PLGA microsphere formulation are necessary for achieving the prolonged (60 h) nerve blockade. The microspheres alone (MLM‑NM) provide a moderate prolongation of approximately 24 hours, while free lidocaine with or without a magnetic field produces only short‑lasting effects (~8 hours).

### Behavioural evaluation

**Fig. 6:**
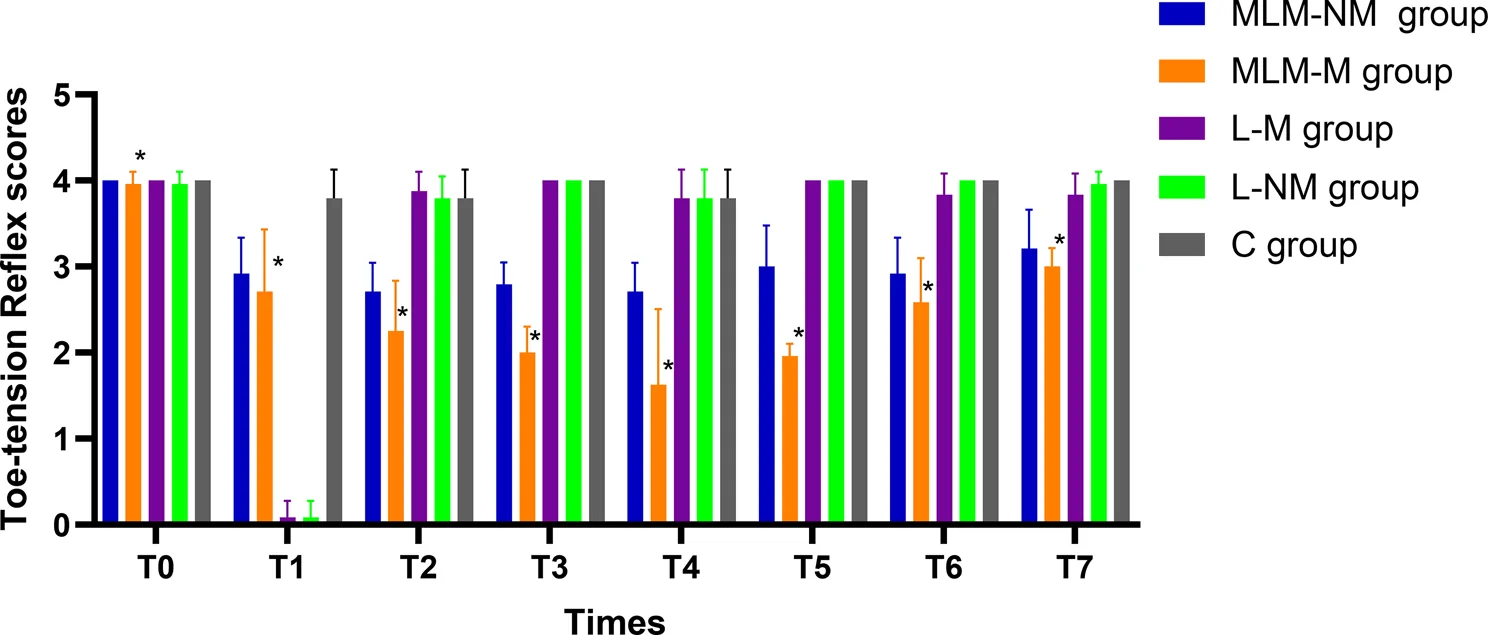
Behavioral assessment of motor function using the Toe‑Opening reflex score.Motor function of the rabbit Hind limb was evaluated at baseline (T0, before injection) and at 0.5 h (T1), 2 h (T2), 8 h (T3), 16 h (T4), 24 h (T5), 48 h (T6), and 60 h (T7) post‑injection. Rabbits were randomly assigned to four groups (*n* = 5 per group): MLM‑M group (magnetic lidocaine microspheres + static magnetic field, 120 mT), MLM‑NM group (magnetic lidocaine microspheres without magnetic field), L‑M group (1% lidocaine solution + static magnetic field), L‑NM group (1% lidocaine solution without magnetic field), and C group (contralateral side, 2 mL saline, no magnetic field). Data are presented as median with interquartile range (IQR). Statistical comparisons among groups at each time point were performed using the Kruskal‑Wallis test with Dunn’s post hoc test for between‑group comparisons at each time point

The toe‑opening reflex scores (Fig. 6) showed a similar trend. The MLM‑M group exhibited a profound motor block (score 0–1) lasting up to 48 hours, with only partial recovery at 60 hours. The MLM‑NM group recovered to near‑baseline function by 24 hours, and the L‑M group recovered by 8 hours. Significant differences were observed between MLM‑M and MLM‑NM at time points 16–60 hours (*p* < 0.05, Kruskal‑Wallis test with Dunn’s post hoc). The L-NM group showed no significant difference from the L-M group at any time point (*p* > 0.05).These functional data corroborate the electrophysiological findings, confirming that magnetic retention is essential for extending the block beyond 24 hours.

### Pharmacodynamic and pharmacokinetic result

**Fig. 7:**
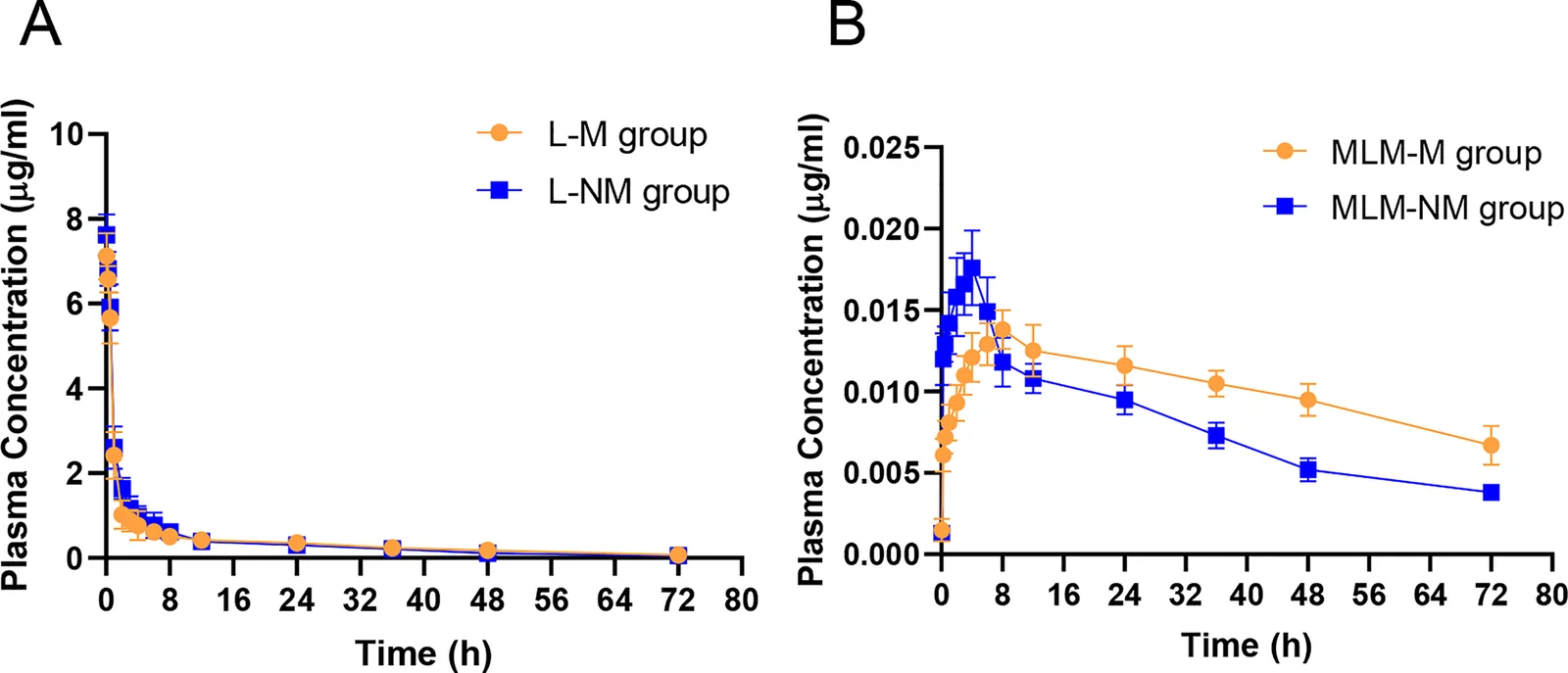
Plasma concentration-time profiles. **A**, plasma concentration-time curves for the lidocaine magnetic field (L-M) and lidocaine (L-NM) groups. **B**, plasma concentration-time curves for the magnetic lidocaine microspheres with an applied magnetic field (MLM-M) group and magnetic lidocaine microspheres (MLM-NM) groups

The blood concentration-time profiles of the groups are shown in Fig. 7, Other pharmacokinetic parameters are shown in Table 1. The MLM-M group prolonged t_1/2_, T_max_, and MRT in the presence of a magnetic field compared with other groups (*p* < 0.05), and in the presence of a magnetic field, Tmax increased about 1.78-fold, t_1/2_ increased about 2.92-fold, and MRT increased about 2.67-fold in the MLM-M group compared with the MLM-NM group. Compared with the L-M and L-NM groups, the MLM-M group showed an increase of about 21.33-fold in Tmax, about 15.62-fold in t1/2, and about 19.59-fold in MRT. The above results indicated that magnetic lidocaine microspheres could maintain the blood concentration in vivo for a longer period of time and prolong the action time of lidocaine to a certain extent. The AUC0-48 and AUC_0-∞_values of the MLM-M group (0.341 ± 0.034 and 0.553 ± 0.082 μg·h/mL, respectively) were approximately 4.5% and 7.4% of the corresponding values in the L-M group (7.566 ± 0.302 and 7.459 ± 0.280 μg·h/mL, *p* < 0.001), reflecting the substantial reduction in systemic lidocaine exposure achieved by the sustained-release microsphere formulation combined with magnetic targeting. Similarly, the MLM-NM group showed significantly lower systemic exposure compared to the free lidocaine groups, though the reduction was less pronounced than in the MLM-M group. This may reflect a significant reduction in the blood entry of lidocaine by the slow release of microspheres.

**Table.1:** Pharmacokinetic parameters （$\bar x \pm s$, *n* = 20）

	MLM-M Group	MLM-NM Group	L-M Group	L-NM Group
*t*_*1/2*_(h)	34.3621 ± 4.1047	11.7707 ± 2.6344^a^	2.4350 ± 0.6743^ab^	2.0212 ± 0.6135^ab^
*T*_max_(h)	6.4000 ± 0.8944	3.6000 ± 0.5477^a^	0.3000 ± 0.1118^ab^	0.3000 ± 0.1118^ab^
*C*_max_(μg/ml)	0.0134 ± 0.0019	0.0176 ± 0.0015	6.5472 ± 0.4372^ab^	6.3979 ± 0.7118^ab^
MRT(h)	49.9505 ± 5.5070	18.7220 ± 1.6319^a^	2.6136 ± 0.2730^ab^	2.4855 ± 0.2408^ab^
AUC_0–48_(μg·h/ml)	0.3408 ± 0.0335	0.2835 ± 0.0460	7.5660 ± 0.3024^ab^	7.6429 ± 0.4723^ab^
AUC_0-∞_(μg·h/ml)	0.5533 ± 0.0818	0.3039 ± 0.0490	7.4585 ± 0.2799^ab^	7.5349 ± 0.4492^ab^

a, compared with MLM-M group, *p* < 0.05; b, compared with L-M group, *p* < 0.05

### Drug distribution in the vivo as shown by MRI

**Fig. 8:**
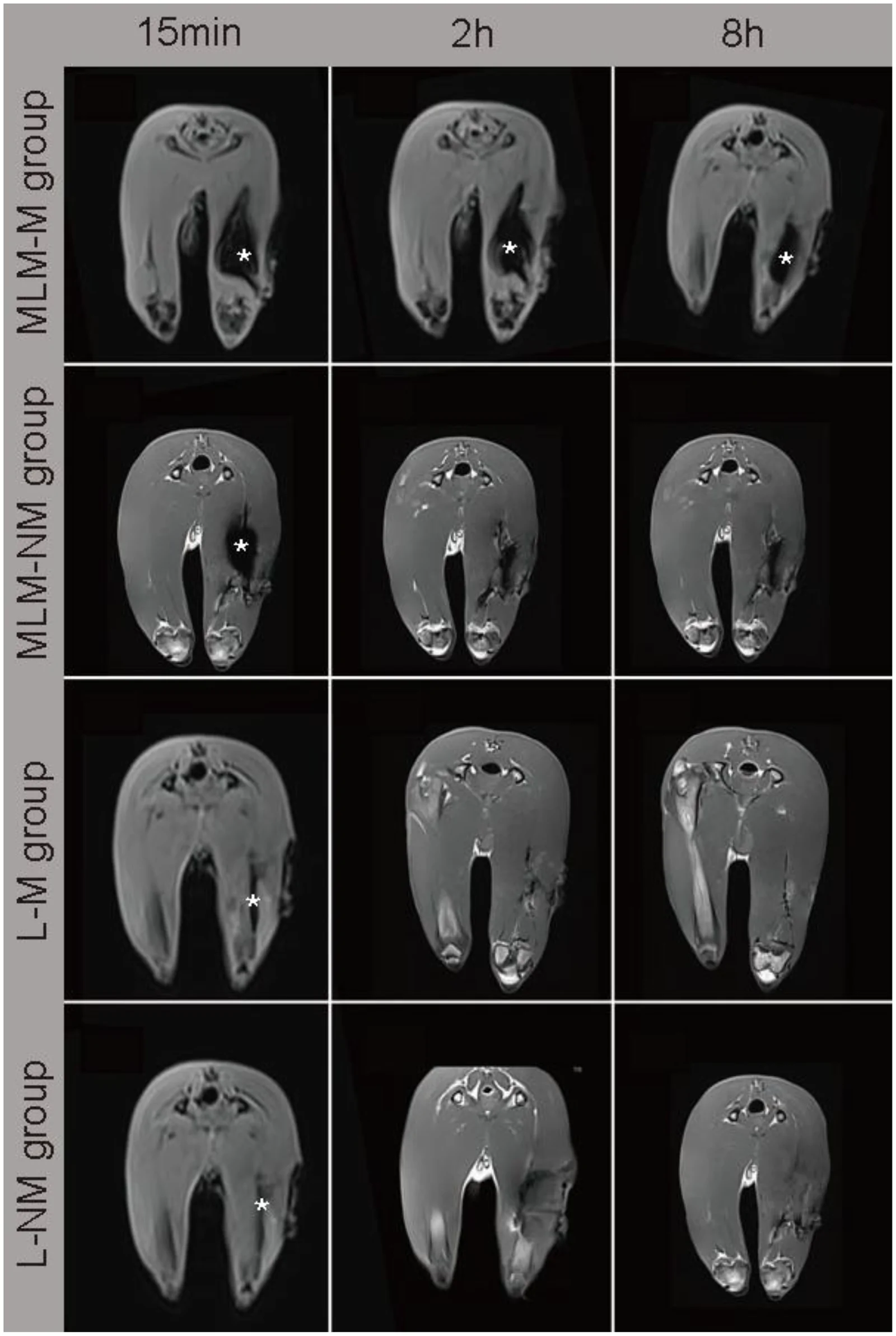
MRI images of the distribution of drugs injected into the periphery of the rabbit sciatic nerve. The image displays MRI scans from four experimental groups (L-NM, L-M, MLM-NM, MLM-M) at three time intervals (15 min, 2 h, 8 h). The white star symbol indicate the location and morphology of the primary drug depot. The MLM-M group shows minimal diffusion of the microsphere depot throughout the 8-hour period. In contrast, lidocaine groups (L-NM group and L-M group) exhibit rapid and extensive diffusion, with the microsphere signal largely dissipated by 8 hours. The MLM-NM group shows an intermediate pattern, with gradual diffusion over time

**Fig. 9:**
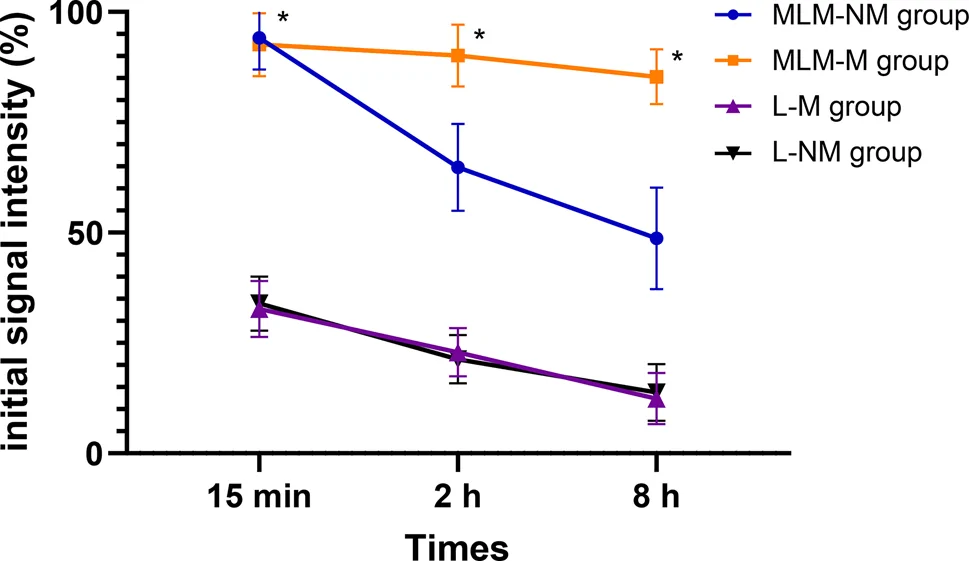
Semi-quantitative analysis of MRI signal intensity over time. Box plots show the percentage of initial signal intensity (relative to baseline) retained within the region of interest (ROI) at 15 min, 2 h, and 8 h after perineural injection. Groups: MLM-M (magnetic lidocaine microspheres + 120 mT static magnetic field), MLM-NM (microspheres without magnetic field), L-M (1% lidocaine solution + magnetic field), and L-NM (lidocaine solution alone). Boxes represent the interquartile range (IQR), horizontal lines inside boxes indicate the median, whiskers denote the 5th–95th percentiles, and individual points show each animal’s data (*n* = 5 per group). In the MLM-M group, signal intensity remained stable over 8 h, with 85.3 ± 6.2% (mean ± SD) of the initial signal retained at 8 h, indicating minimal microsphere dispersion. The MLM-NM group showed progressive signal loss, retaining only 48.7 ± 11.5% at 8 h (*p* < 0.01 vs. MLM-M). Both lidocaine groups (L-M and L-NM) exhibited rapid signal decline, with the L-M group retaining only 12.4 ± 5.8% at 8 h (*p* < 0.001 vs. MLM-M). These results demonstrate that magnetic targeting (MLM-M) significantly improves local drug retention compared to non-magnetic microspheres or free lidocaine

Figure 8 presents representative MRI images of all experimental groups. Figure 9 shows a Semi-quantitative analysis of time-dependent signal intensity within the region of interest (ROI) revealed distinct local retention and diffusion behaviors among different groups. In the MLM-M group, no evident local diffusion was observed at 15 min, 2 h, and 8 h post-administration, with microsphere signal remaining largely unchanged in size. This group retained 85.3 ± 6.2% of the initial signal intensity at 8 h. By contrast, the MLM-NM group exhibited mild diffusion at 2 h and markedly enhanced diffusion at 8 h, accompanied by gradual shrinkage of microsphere signal, with only 48.7 ± 11.5% of the initial signal intensity preserved at 8 h (*p* < 0.01 vs. MLM-M group). Furthermore, both the L-M and L-NM groups displayed prominent local diffusion as early as 15 min after drug administration, which further intensified at 2 h. By 8 h, microsphere signal were barely detectable at the local administration site. Specifically, the L-M group retained merely 12.4 ± 5.8% of the initial signal intensity at 8 h (*p* < 0.001 vs. MLM-M group), demonstrating that magnetic targeting significantly improves the local retention of the drug.

### Sciatic nerve pathology results

Histological sections from the MLM-M and L-M groups were evaluated using a semi-quantitative scoring system assessing four parameters: inflammatory cell infiltration, perineural fibrosis, axonal degeneration, and myelin sheath integrity (each scored 0–3; see Methods for detailed criteria). All assessments were performed by two independent pathologists under blinded conditions, with substantial inter-rater agreement (Cohen’s κ = 0.79–0.88; ICC = 0.87–0.93 for all parameters).

**Fig. 10:**
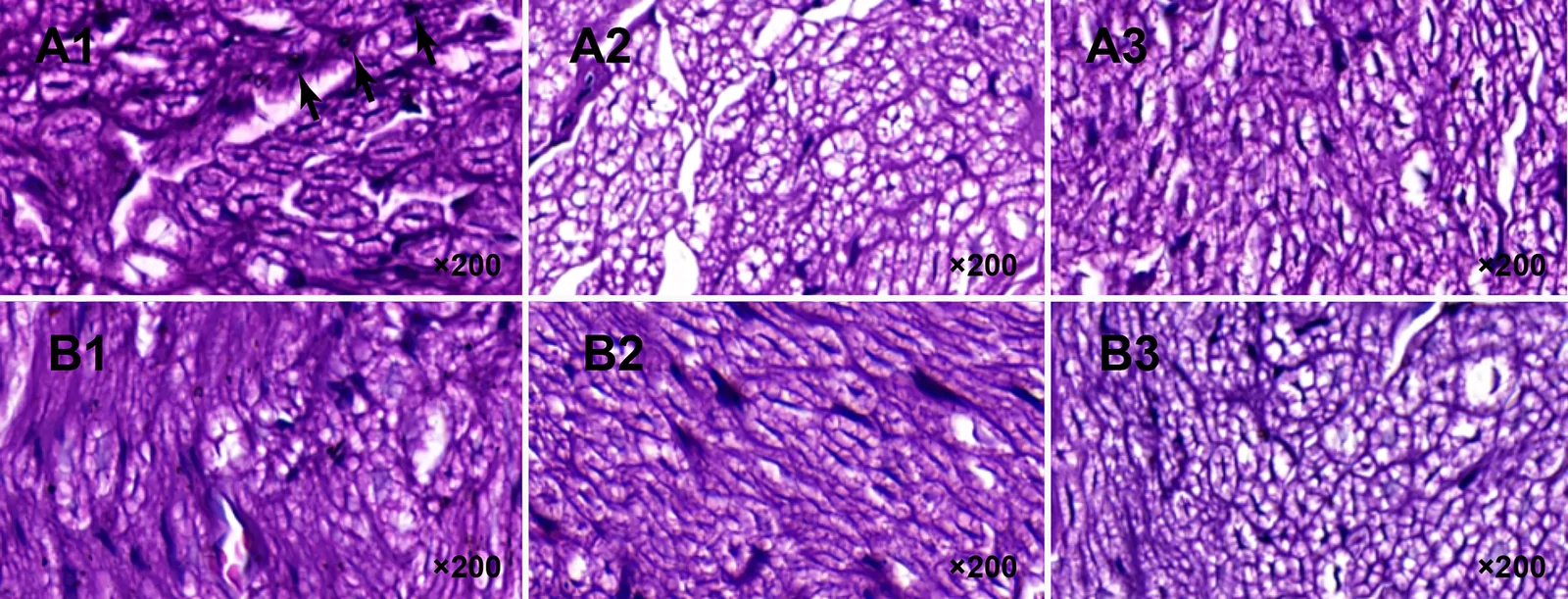
Pathological sections of rabbit sciatic nerve after perineural injection. (A1, A2, A3) Representative hematoxylin and eosin (H&E) stained sections from the MLM-M group (injected with magnetic lidocaine microspheres under a static magnetic field) at 3, 7, and 14 days post-injection, respectively. (B1, B2, B3) Representative H&E stained sections from the L-M group (injected with plain lidocaine solution under a static magnetic field) at 3, 7, and 14 days post-injection, respectively.respectively.The nerve tissue structure remains intact in all sections. In the MLM-M group, a minimal and transient inflammatory cell infiltration (indicated by black arrowheads) is observed in the perineural tissue at day 3 (A1), which resolves by days 7 (A2) and 14 (A3). In contrast, the L-M control group shows no significant inflammatory cell infiltration at any of the observed time points (B1–B3)

The quantitative scores are summarized in Table 2 and representative images are shown in Fig. 10. In the MLM-M group, inflammatory cell infiltration was mild at Day 3 (score 1.2 ± 0.4), decreased significantly by Day 7 (0.3 ± 0.5, *p* < 0.05 vs. Day 3), and was comparable to baseline by Day 14 (0.1 ± 0.3). No significant perineural fibrosis, axonal degeneration, or myelin disruption was observed at any time point in this group (all scores ≤ 0.3). In the L-M group, all histological parameters remained near-baseline levels throughout the observation period, with inflammatory scores of 0.2 ± 0.4 at Day 3, 0.1 ± 0.3 at Day 7, and 0.0 ± 0.0 at Day 14. Two-way ANOVA revealed a statistically significant difference in inflammatory score between the MLM-M and L-M groups at Day 3 only (*p* = 0.032); no significant between-group differences were detected at Days 7 or 14 for any parameter (all *p* > 0.05).

**Table 2:** Semi-quantitative histological scores of rabbit sciatic nerve and perineural tissue （$\bar x \pm s$, *n* = 2）

Parameter	Group	Day 3	Day 7	Day 14
Inflammatory cell infiltration	MLM-M	1.2 ± 0.4	0.3 ± 0.5*	0.1 ± 0.3
	L-M	0.2 ± 0.4	0.1 ± 0.3	0.0 ± 0.0
Perineural fibrosis	MLM-M	0.3 ± 0.5	0.2 ± 0.4	0.1 ± 0.3
	L-M	0.1 ± 0.3	0.1 ± 0.3	0.0 ± 0.0
Axonal degeneration	MLM-M	0.1 ± 0.3	0.0 ± 0.0	0.0 ± 0.0
	L-M	0.0 ± 0.0	0.0 ± 0.0	0.0 ± 0.0
Myelin sheath integrity	MLM-M	0.2 ± 0.4	0.1 ± 0.3	0.0 ± 0.0
	L-M	0.1 ± 0.3	0.0 ± 0.0	0.0 ± 0.0

*p < 0.05 versus L‑M group at Day 3. Statistical analysis: Two‑way ANOVA with Tukey’s post hoc test. Inter‑rater reliability: Cohen’s weighted Kappa = 0.86 (95% CI: 0.79–0.93)

These findings indicate that magnetic lidocaine microspheres induced only mild, transient perineural inflammation that resolved within one week, with no evidence of progressive fibrosis, axonal injury, or demyelination during the 14-day observation period. These descriptive findings suggest a transient local response, but should be interpreted with caution given the limited sample size (*n* = 2 per time point).

### CCK-8 results

**Fig. 11:**
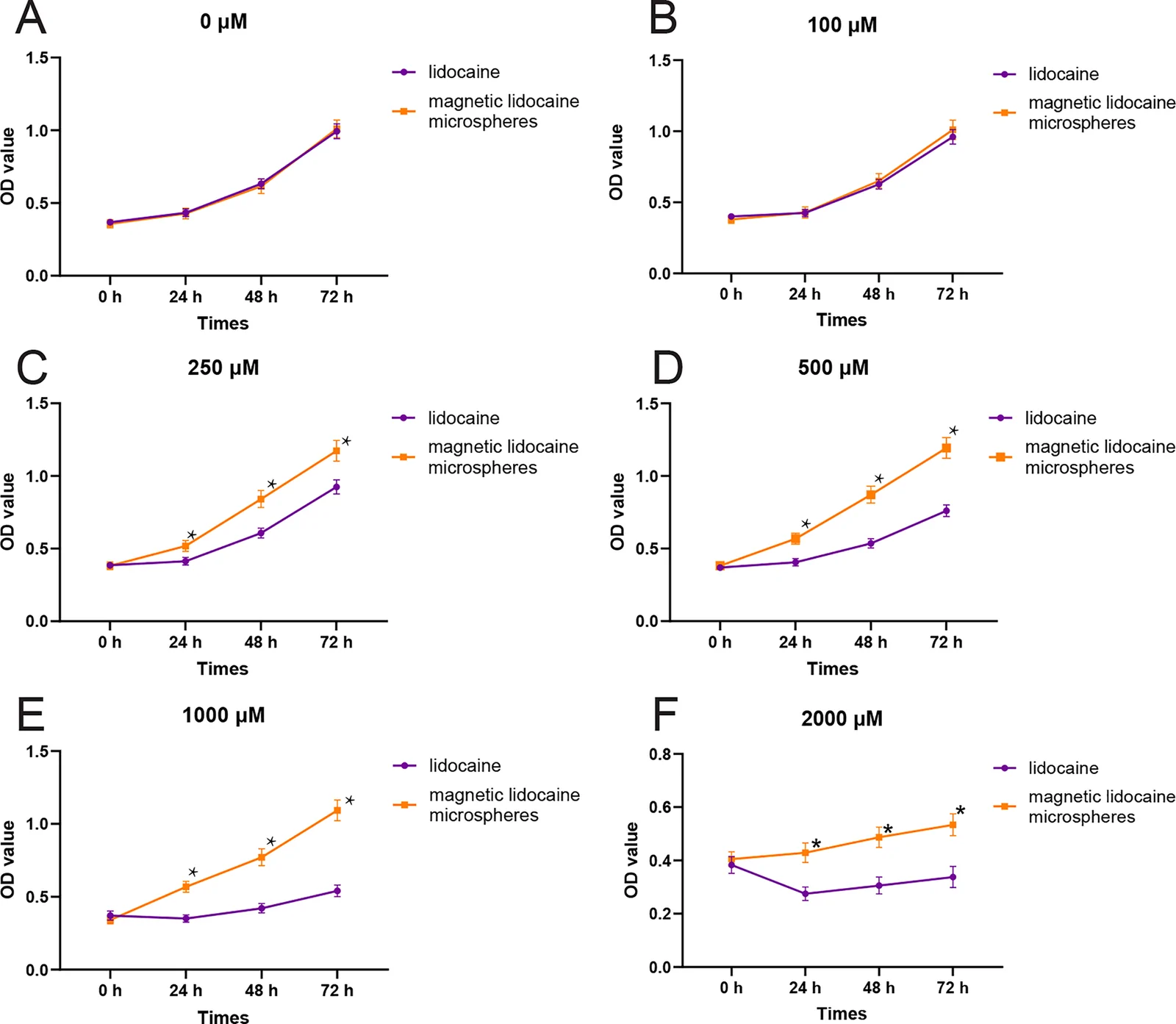
Cytotoxicity assessment of lidocaine and magnetic lidocaine microspheres (MLM) on rat Schwann cells (RSC96 cell line).Cell viability was measured using the CCK-8 assay after 72 hours of exposure to various concentrations (0, 100, 250, 500, 1000, or 2000 μM) of either free lidocaine (**A**, **B**, **C**) or an equivalent concentration of lidocaine encapsulated in magnetic microspheres (**D**, **E**, **F**). Data are expressed as percentage viability relative to the untreated control group (0 μM). Free lidocaine exhibits a clear dose-dependent cytotoxicity, with significant reduction in cell viability observed at 250 μM and above. In contrast, treatment with magnetic lidocaine microspheres (MLM) resulted in significantly higher cell viability across all corresponding concentrations, demonstrating that the microsphere formulation reduced lidocaine‑induced cytotoxicity, likely due to the sustained release profile that lowers instantaneous free drug concentrations

The results of the CCK-8 assay are shown in Fig. 11. Free lidocaine induced a clear dose-dependent reduction in Schwann cell viability, with statistically significant cytotoxicity observed at concentrations ≥ 250 μM compared to untreated controls (*p* < 0.01). At concentrations of 500 μM and below, cells treated with magnetic lidocaine microspheres showed significantly higher viability compared to those treated with equivalent concentrations of free lidocaine (*p* < 0.05 at 500 μM; *p* < 0.01 at 250 μM), demonstrating that encapsulation in microspheres reduced the cytotoxic impact of lidocaine, likely by limiting the instantaneous free drug concentration through sustained release. At the highest concentration tested (2000 μM), both formulations resulted in markedly reduced viability, though the microsphere group maintained a modest but significantly higher viability (*p* < 0.05).

## Discussion

Addressing the critical need for extended-duration local anesthetics that optimize both therapeutic effect and safety, this investigation presents a novel magnetic lidocaine microsphere system composed of PLGA and superparamagnetic iron oxide nanoparticles. When deployed with an applied magnetic field, these microspheres demonstrate sustained, site-specific lidocaine release maintaining effective nerve blockade for over 60 hours. Importantly, the platform achieves this prolonged analgesia while significantly reducing systemic drug exposure compared to conventional lidocaine formulations, as evidenced by lower peak plasma concentrations and diminished total circulatory load. Safety assessments reveal only minimal, transient perineural inflammation and substantially decreased Schwann cell cytotoxicity relative to lidocaine.

Management of postoperative pain remains one of the most challenging aims of modern anesthesia. Opioids have been the mainstay of postoperative pain management for decades; however, they may also lead to adverse events, e.g., respiratory depression, tolerance and hyperalgesia. Regional anesthesia techniques have shown their benefit by reducing opioid use and shortening the stay in the postanesthesia care units, thus local anesthetics represent an important alternative to the use of systemic opioids [4].

An ideal local anesthetic should have (1) rapid onset of dense blockade, permitting surgery under local or regional anesthesia [8, 9]; (2) persistence of dense and reliable blockade more than 24 h,^8^ and (3) a prolonged period of partial blockade over the next 2 or 3 days [10]. Since the synthesis of lidocaine in 1943, numerous approaches have been developed for prolonging the duration of analgesia. Among these, liposomal bupivacaine (Exparel®) represents the most widely used extended-release local anesthetic formulation in current clinical practice, with a labeled duration of up to 72 hours. However, recent systematic reviews have demonstrated that while liposomal bupivacaine provides a modest reduction in rest pain scores at 24–72 hours compared to plain bupivacaine, it has not consistently shown significant reductions in opioid consumption. Furthermore, liposomal bupivacaine relies on passive diffusion from the injection site and cannot be actively targeted or retained at specific nerve locations. These limitations highlight the continued need for novel drug delivery systems that can achieve both prolonged and actively targeted local anesthetic effects. Perineural and wound catheter infusions can extend the duration of analgesia. Conversely, catheters are sometimes more cumbersome to place compared with single-shot blocks, can dislodge and require tethering the patient to a pump. These limitations promoted the researchers to seek new long-acting local anesthetics.

The clinical application of local anesthetics has been optimized through modifications in administration techniques and concentrations, as well as the incorporation of adjuncts to expedite the onset and prolong the duration of analgesia [3]. While liposomal bupivacaine can extend the duration of analgesia beyond that of conventional local anesthetics, its clinical performance has been inconsistent, and the duration of most widely available local anesthetics typically does not surpass 24 hours. Consequently, there remains a need to identify safe and reliable methods for more precise drug targeting to maximize the duration of local anesthetic efficacy while minimizing drug dosage and mitigating toxic side effects [1, 11]. Based on the dose-dependent nature of local anesthetics, some scholars have started to develop local anesthetic in drug delivery systems (LA-in-DDS) to achieve slow or on-demand release of drugs, which has the advantage of reducing the accumulation of drugs in the body and achieving sustained analgesic effects at lower doses/concentrations. [12–14]. The advantage of LA-in-DDS is that it reduces drug accumulation in the body and achieves sustained analgesia at lower doses/concentrations. Currently, novel drug delivery systems include implantable drug delivery systems (e.g., microchips, transdermal patches, etc.), injectable liquids (e.g., hydrogels, etc.), and injectable micron- or nanoscale drug delivery systems (e.g., PLA, PLGA, etc.) [15]. Drug delivery systems with sustained-release characteristics have considerable potential to improve patient compliance, reduce the frequency of administration and enhance efficacy, and the development of new drug delivery systems in terms of prolonging the duration of action has a greater potential for application. SABER-bupivacaine is a biodegradable lipophilic viscous liquid that can be injected intramuscularly and subcutaneously.Prabhakar [16] reviewed experimental studies on SABER-bupivacaine and found that SABER-bupivacaine was effective in reducing the use of opioids as compared to SABER-placebo.HTX-011 is an novel formulation of extended-release bupivacaine compounded with meloxicam, which are co-loaded in a corrosive polymer [17]. In two completed phase III trials on HTX-011, patients using HTX-011 experienced a sustained decrease in pain perception over 72 h and a decrease in postoperative opioid use [18, 19]. In an open inguinal hernia repair, patients given HTX-011 had the potential to reduce opioid use with higher satisfaction [20, 21]. Synthesised a sustained-release system of lidocaine polymerised hydrogel local anaesthetics, and experimental results demonstrated that the formulation had an anaesthetic effect, with a sustained in vitro release up to 60 h.^21^

In our study, we encapsulated lidocaine within PLGA to create nano-scale PLGA-lidocaine microspheres, a synthetic polymer known for its excellent histocompatibility and biodegradability [22, 23]. The metabolic byproducts of PLGA are benign, consisting of water and carbon dioxide [14, 24], thereby precluding any toxic side-effects in medical and biomaterial applications. Our findings revealed that the magnetic lidocaine microspheres demonstrated favorable sustained-release characteristics in both in vitro assays and animal models. In vitro, the cumulative lidocaine release reached 76% within the initial 24-hour burst release phase and 97% by 60 hours. This compelling evidence establishes that the magnetic lidocaine microspheres possess a duration of action exceeding 60 hours, offering a superior, extended analgesic effect in comparison to conventional lidocaine, which is extensively utilized in current clinical settings. Based on previous studies [25], subsequent to the synthesis of magnetic lidocaine microspheres, we incorporated iron oxide nanoparticles, known for their magnetic responsiveness, into the PLGA to confer magnetic targeting capabilities upon the PLGA-lidocaine microspheres, thereby creating magnetic lidocaine microspheres. The objective is to immobilize the magnetic lidocaine microspheres at the injection site using an applied static magnetic field, thereby preventing their systemic absorption and subsequent loss of efficacy. Additionally, our study demonstrated that the magnetic lidocaine microspheres exhibited robust magnetic responsiveness in in vitro settings and superparamagnetic behavior in animal models; specifically, in the presence of an applied static magnetic field, they aggregated at the injection site over an extended period, as evidenced by the distinct blackening of the injection site in MRI scans.

The blood concentration assay outcomes indicated that the AUC_0–48_ and AUC_0-∞_ values for magnetic lidocaine microspheres were approximately 22-fold and 13-fold lower than those for lidocaine, respectively, under the influence of an applied static magnetic field. The magnetic lidocaine microspheres were distinguished by reduced peak concentrations, a gradual increase in blood levels, and a retarded onset of peak concentrations. This implicates that, with a peripheral magnetic field, the microspheres are sequestered around the nerves, minimally penetrating the bloodstream. Concurrently, the sustained release of the microspheres significantly diminishes the lidocaine entering the systemic circulation, thereby enhancing the safety profile of this local anesthetic to a certain degree. The safety profile of extended-release analgesics has likewise been a central concern in clinical practice. Preliminary pathological examination suggested that magnetic lidocaine microspheres induced only minimal local inflammatory cell infiltration in the limited samples examined (*n* = 2 per time point). While these descriptive findings are encouraging, they should be confirmed in future studies with adequate statistical power.

Our comparative analysis reveals that the prolonged nerve blockade seen in the MLM‑M group is the result of a synergistic effect between the PLGA sustained‑release microspheres and the external magnetic field. The MLM‑NM group (microspheres without magnetic field) produced a block lasting approximately 24 hours, which is longer than free lidocaine (L‑M group, ~8 hours), indicating that the microsphere encapsulation alone extends the duration. Critically, the addition of the magnetic field (MLM‑M) further extended the block to 60 hours, demonstrating the value of magnetic retention in preventing microsphere dispersion. These findings establish that neither component alone can achieve the ultra‑long block observed in the full system.

The extended nerve blockade, markedly reduced systemic exposure, and improved targeting retention observed via MRI in our study are primarily attributable to the high magnetic field gradient (6 T/m) generated by the Halbach array. The magnetic force exerted on the superparamagnetic microspheres is proportional to this gradient. Thanks to their high saturation magnetization (~1.0 emu/g), the microspheres developed in this work experience sufficient magnetic force under a 6 T/m gradient to counteract dissipative forces—such as diffusion within the interstitial fluid and scouring from local microcirculatory flow—effectively retaining them at the injection site. These findings suggest that, rather than simply increasing magnetic field strength, optimizing the magnetic field gradient is more critical for achieving efficient targeting at relatively low field strengths (120 mT). This “low-field-strength, high-gradient” approach not only ensures effective targeting but also avoids the clinical limitations and potential risks associated with large high-field-strength magnets—a significant advantage for future clinical translation.

Furthermore, cellular assays confirmed that lidocaine exerted a direct cytotoxic effect on Schwann cells in neonatal mice, with a marked decrease in the number of deceased Schwann cells following treatment with magnetic lidocaine microspheres as opposed to lidocaine alone. This phenomenon is most likely attributable to the sustained‑release characteristics of the microsphere formulation, which reduce the instantaneous free lidocaine concentration to which the cells are exposed, rather than reflecting any intrinsic cytoprotective property of the carrier materials. It is also important to note that the concentrations reported for the microsphere treatment groups refer to lidocaine equivalents; the actual free lidocaine concentration in the culture medium at any given time is substantially lower due to the sustained release profile. Therefore, the observed improvement in cell viability should be interpreted as a reduction in cytotoxicity conferred by sustained release, rather than as an active protective effect of the microspheres. Future studies using blank microspheres (without lidocaine) would help distinguish the effects of the carrier materials from those of the released drug. The rat Schwann cell line (RSC96) was selected as a well-established and widely used model for studying peripheral neurotoxicity in vitro. Although RSC96 cells retain key phenotypic features of primary Schwann cells, including S100β expression, it should be noted that immortalized cell lines may exhibit different sensitivities to pharmacological agents compared to primary cells. Therefore, the cytotoxicity results should be interpreted with this consideration, and confirmation with primary Schwann cell cultures would strengthen future studies.

We acknowledge that the present study did not include a blank microsphere control (PLGA/Fe₃O₄ microspheres without lidocaine), which would have allowed us to definitively distinguish the cytotoxicity attributable to the carrier materials from that induced by lidocaine itself. However, the safety and biocompatibility of PLGA as a drug delivery carrier are extensively documented. PLGA is an FDA‑approved biodegradable polymer, and its degradation products (lactic acid and glycolic acid) are metabolized via natural physiological pathways. Multiple independent studies have confirmed that blank PLGA microspheres and nanoparticles exhibit no significant cytotoxicity across a range of cell types and concentrations [25, 26], , and are well tolerated in vivo with only mild, transient tissue responses. Nevertheless, we agree that future studies incorporating blank microsphere controls would provide direct comparative data within our specific experimental system and further strengthen the safety assessment.

Additionally, the rabbit sciatic nerve is anatomically more superficial than the human sciatic nerve, which could potentially facilitate more effective magnetic targeting in the animal model than would be achievable in clinical settings. Future translational studies should evaluate the efficacy of this approach in large-animal models with deeper nerve targets to better assess clinical feasibility. Furthermore, the in vitro release studies were conducted in PBS (pH 7.4) at 37 °C, which does not fully recapitulate the complex perineural tissue environment that includes protein binding, enzymatic activity, and local vascular clearance. These factors may influence the in vivo release kinetics and should be investigated in future studies using tissue-simulating media. We acknowledge that the 14‑day observation period was chosen to capture the acute‑to‑subacute local response, which typically resolves within two weeks. Our histological findings showed only mild, transient inflammation that resolved by day 14, consistent with previous reports that PLGA microspheres evoke a mild, localized, and self‑limiting inflammatory response that is well tolerated. However, the mild inflammatory cell infiltration observed in the MLM‑M group at day 3 could be attributable to the PLGA/Fe₃O₄ carrier itself rather than to lidocaine. The absence of an empty carrier control (microspheres without lidocaine + magnetic field) limits our ability to definitively attribute this response. Furthermore, the non‑biodegradable Fe₃O₄ core requires longer‑term evaluation. The PLGA shell will gradually release Fe₃O₄ particles, which may be phagocytosed and transported systemically. We have not conducted follow‑up beyond 14 days, nor have we assessed systemic iron burden or organ function. Therefore, future studies should include blank microsphere controls and extend the observation period to at least 6 months to evaluate nerve recovery, carrier‑related inflammation, iron clearance, and systemic safety parameters, in order to comprehensively validate the long‑term safety of this system.

## Conclusion

In summary, this preclinical study provides initial evidence that magnetic lidocaine microspheres, under a static magnetic field, can sustain sciatic nerve blockade for over 60 h while markedly lowering systemic lidocaine exposure and eliciting only minimal, self‑limiting perineural inflammation in rabbits. These findings suggest that magnetically guided, sustained‑release local anesthetics hold promise for prolonged regional analgesia. Nevertheless, because this proof‑of‑concept work did not include drug‑free carrier controls or long‑term (≥6 months) safety monitoring, the observed local tissue response cannot be unequivocally attributed solely to lidocaine, and the long‑term fate of the non‑biodegradable Fe₃O₄ core remains unknown. Future studies should incorporate postoperative pain models, larger animals, and comprehensive evaluations of nerve regeneration, iron clearance, and organ function to establish the translational feasibility and overall risk‑benefit profile of this system.

## Data Availability

The data that support the findings of this study are available from the corresponding author upon reasonable request. Some data are not publicly available due to a pending patent application. All other relevant data are included in the manuscript.

## Abbreviations

ANOVA: Analysis of Variance
AUC: Area Under the Curve
CCK-8: Cell Counting Kit-8
C_max_: Maximum concentration
DCM: Dichloromethane
EDTA: Ethylenediaminetetraacetic acid
Fe₃O₄: Nano-iron oxide (magnetite)
HE: Hematoxylin and eosin
IQR: Interquartile range
LC-MS: Liquid chromatography-mass spectrometry
MRT: Mean residence time
MRI: Magnetic resonance imaging
NaOH: Caustic soda / Sodium hydroxide
PBS: Phosphate buffered saline
PLGA: Poly(lactic-co-glycolic acid)
PVA: Polyvinyl alcohol
ROI: Region of interest
RSC96: Rat Schwann cell line
SD: Standard deviation
SEM: Scanning electron microscopy
TEM: Transmission electron microscopy
Tmax: Time to reach maximum concentration
t_₁/₂_: Elimination half-life
UV: Ultraviolet
VSM: Vibrating sample magnetometer

## References

[1] Desai N, Kirkham KR, Albrecht E. Local anaesthetic adjuncts for peripheral regional anaesthesia: a narrative review. Anaesthesia. 2021;76(S1):100–09. 10.1111/anae.15245. 33426668

[2] Bowness J, Taylor A. Ultrasound-guided regional anaesthesia: visualising the nerve and needle. Adv Exp Med Biol. 1235;2020:19–34. 10.1007/978-3-030-37639-0_2 32488634

[3] Krishna Prasad GV, Khanna S, Jaishree SV. Review of adjuvants to local anesthetics in peripheral nerve blocks: current and future trends. Saudi J Anaesth. 2020;14(1):77–84. 10.4103/sja.SJA_423_19. PMC6970354 31998024

[4] Gohad R, Jain S. Regional anaesthesia, contemporary techniques, and associated advancements: a narrative review. Cureus. 2024;16:e65477. 10.7759/cureus.65477. PMC11346749 39188450

[5] Bassand C, Freitag J, Benabed L, Verin J, Siepmann F, Siepmann J. PLGA implants for controlled drug release: impact of the diameter. Eur J Pharm Biopharmaceutics. 2022;177:50–60. 10.1016/j.ejpb.2022.05.020. 35659920

[6] Singh S, Singha P. Effect of modifications in poly (lactide-co-glycolide) (PLGA) on Drug release and degradation characteristics: a mini review. CDD. 2021;18(10):1378–90. 10.2174/1567201818666210510165938. 33970845

[7] Su Y, Zhang B, Sun R, Liu W, Zhu Q, Zhang X, et al. PLGA-based biodegradable microspheres in drug delivery: recent advances in research and application. Drug Deliv. 2021;28(1):1397–418. 10.1080/10717544.2021.1938756. PMC8248937 34184949

[8] Oda Y. Development of long-acting local anesthetics: a long way from basic research to clinical application. J Anesth. 2024;38(5):581–83. 10.1007/s00540-024-03387-5. 39120639

[9] Zhang W, Wu M, Shen C, Wang Z, Zhou X, Guo R, et al. A new long-acting analgesic formulation for postoperative pain management. Int J Pharm. 2024;664:124599. 10.1016/j.ijpharm.2024.124599. 39154917

[10] Wei Y, Wu Y, Wen K, Bazybek N, Ma G. Recent research and development of local anesthetic-loaded microspheres. J Mater Chem B. 2020;8(30):6322–32. 10.1039/D0TB01129K. 32614031

[11] Nguyen A, Grape S, Gobbetti M, Albrecht E. The postoperative analgesic efficacy of liposomal bupivacaine versus long-acting local anaesthetics for peripheral nerve and field blocks: a systematic review and meta-analysis, with trial sequential analysis. Eur J Anaesthesiol. 2023;40(9):624–35. 10.1097/EJA.0000000000001833. PMC10860892 37038770

[12] Guo W, Cao D, Rao W, Sun T, Wei Y, Wang Y, et al. Achieving long-acting local analgesia using an intelligent hydrogel encapsulated with drug and pH regulator. ACS Appl Mater Interface. 2023;15(36):42113–29. 10.1021/acsami.3c03149. 37639647

[13] Hou Y, Meng X, Zhang S, Sun F, Liu W. Near-infrared triggered ropivacaine liposomal gel for adjustable and prolonged local anaesthesia. Int J Pharm. 2022;611:121315. 10.1016/j.ijpharm.2021.121315. 34826592

[14] Li X, Wei Y, Wen K, Han Q, Ogino K, Ma G. Novel insights on the encapsulation mechanism of PLGA terminal groups on ropivacaine. Eur J Pharm Biopharmaceutics. 2021;160:143–51. 10.1016/j.ejpb.2021.01.015. 33524537

[15] Butreddy A, Gaddam RP, Kommineni N, Dudhipala N, Voshavar C. PLGA/PLA-Based long-acting injectable depot microspheres in clinical use: production and characterization overview for Protein/Peptide delivery. IJMS. 2021;22(16):8884. 10.3390/ijms22168884. PMC8396256 34445587

[16] Prabhakar A, Ward CT, Watson M, Sanford J, Fiza B, Moll V, et al. Liposomal bupivacaine and novel local anesthetic formulations. Best Pract Res Clin Anaesthesiology. 2019;33(4):425–32. 10.1016/j.bpa.2019.07.012. 31791561

[17] Viscusi E, Gimbel JS, Pollack RA, Hu J, Lee GC. HTX-011 reduced pain intensity and opioid consumption versus bupivacaine HCl in bunionectomy: phase III results from the randomized EPOCH 1 study. Reg Anesth Pain Med. 2019, [Epub ahead of print]44(7):700–06. 10.1136/rapm-2019-100531. 31113830

[18] Blair HA. Bupivacaine/Meloxicam prolonged release: a review in postoperative pain. Drugs. 2021;81(10):1203–11. 10.1007/s40265-021-01551-9. 34228280

[19] Cornett EM, Turpin MAC, Busby M, Pham AD, Kallurkar A, Brondeel KC, et al. HTX-011 (bupivacaine and meloxicam) for the prevention of postoperative pain – clinical considerations. Pain Manag. 2021;11(4):347–56. 10.2217/pmt-2020-0097. 33618542

[20] Minkowitz H, Soto R, Fanikos J, Hammer GB, Mehta N, Hu J, et al. Opioid-free recovery after hernia repair with HTX-011 as the foundation of a non-opioid, multimodal analgesia regimen in a real-world setting: a randomized, open-label study. Pain Ther. 2021;10(2):1295–308. 10.1007/s40122-021-00289-2. PMC8586395 34318438

[21] Li Y, Zhao E, Li L, Bai L, Zhang W. Facile design of lidocaine-loaded polymeric hydrogel to persuade effects of local anesthesia drug delivery system: complete in vitro and in vivo toxicity analyses. Drug Deliv. 2021;28(1):1080–92. 10.1080/10717544.2021.1931558. PMC8204985 34114924

[22] Meng Y, Niu X, Li G. Liposome Nanoparticles as a novel Drug delivery system for therapeuticand diagnostic applications. CDD. 2023;20(1):41–56. 10.2174/1567201819666220324093821. 35331112

[23] Yanar F, Carugo D, Zhang X. Hybrid nanoplatforms comprising organic nanocompartments encapsulating inorganic nanoparticles for enhanced drug delivery and bioimaging applications. Molecules. 2023;28(15):5694. 10.3390/molecules28155694. PMC10420199 37570666

[24] Prakash S. Nano-based drug delivery system for therapeutics: a comprehensive review. Biomed Phys Eng Express. 2023;9(5):052002. 10.1088/2057-1976/acedb2. 37549657

[25] Wang Y, Siebzehnrubl D, Weller M, Weiss T, Siebzehnrubl FA, Newland B. Vortioxetine: a potential drug for repurposing for Glioblastoma treatment via a microsphere local delivery system. ACS Biomater Sci Eng. 2025;11(4):2203–15. 10.1021/acsbiomaterials.5c00068. PMC12001186 40167528

[26] Park JH, Cho HJ, Kim DD. Poly((D,L)lactic-glycolic)acid–star glucose nanoparticles for glucose transporter and hypoglycemia-mediated tumor targeting. IJN. 2017;Volume 12(12):7453–67. 10.2147/IJN.S147668. PMC5644567 29066894

